# Effects of a return to work program on the health and barriers to returning to work in head and neck cancer patients: A randomized controlled trial

**DOI:** 10.1016/j.apjon.2023.100320

**Published:** 2023-10-17

**Authors:** Ya-Lan Chang, Bing-Shen Huang, Chien-Yu Lin, Ching-Fang Chung, Shu-Ching Chen

**Affiliations:** a Department of Nursing, Linkou Chang Gung Memorial Hospital, Taoyuan, Taiwan; b Department of Nursing, College of Nursing, Chang Gung University of Science and Technology, Taoyuan, Taiwan; c Department of Radiation Oncology and Proton and Radiation Therapy Center, Linkou Chang Gung Memorial Hospital, Taoyuan, Taiwan; d Department of Medical Imaging and Radiological Sciences, College of Medicine, Chang Gung University, Taoyuan, Taiwan; e School of Nursing and Geriatric and Long-Term Care Research Center, College of Nursing, Chang Gung University of Science and Technology, Taoyuan, Taiwan; f School of Nursing, College of Medicine, Chang Gung University, Taoyuan, Taiwan

**Keywords:** Head and neck cancer, Return to work, Transitional care, Return to work program, Intervention, Survivorship

## Abstract

**Objective:**

The aim of the study was to evaluate the effects of a return to work (RTW) program on perceived health status, barriers to returning to work, fear of cancer progression, social support, physical function, and psychosocial function in head and neck cancer (HNC) patients.

**Methods:**

A randomized controlled trial with repeated measures was conducted. The 70 HNC patients were randomly assigned into two groups: 35 in the experimental group (RTW) and 35 in the control group (usual care). Patients were assessed at four time points: baseline (T0) (6 months after completing treatment), and then at 9-, 12-, and 15-months (T1, T2, and T3, respectively) after completing treatment. Patients completed a self-reported questionnaire, including measures of perceived health status, barriers to returning to work, fear of cancer progression, social support, physical function, and psychosocial function.

**Results:**

Patients in the experimental group had significantly greater perceived health status and better psychosocial function compared to those in the control group. Compared to T0, at T4, participants in both groups had significantly lower levels of barriers to returning to work, fear of cancer progression, social support, and higher levels of physical function.

**Conclusions:**

The RTW program effectively improved perceived health status and psychosocial function in HNC patients. Survivorship care should include a transitional return-to-work program to help patients transition back to work.

**Trial registration:**

NCT04322695.

## Introduction

Globally, cancer remains a leading cause of mortality, accounting for 10 million deaths and an estimated 19.3 million new cases in 2020.^1^ Head and neck cancers (HNCs) are the fifth most common type of cancer, projected to result in nearly 200,000 deaths each year worldwide.^2^ In Taiwan, HNC is the fourth leading cause of cancer death among males.^3^ Most HNC patients are labor workers, chew betel quid, smoke, drink alcohol,^4^ are the family's primary wage earner, and have moderate to low socioeconomic status.^5^, ^6^, ^7^ The adverse effects of treatment on HNC patients may cause physical inactivity, psychosocial distress, and perceived poor health status, resulting in barriers to returning to work (RTW).^5^^,^^6^

Barriers to RTW are part of a complex process that includes a lack of suitable social support, a lack of appropriate jobs, and a lack of up-to-date skills.^8^, ^9^, ^10^ Greidanus et al.^10^ found that cancer survivors reported RTW-related barriers that included support, communication, work environment, discrimination, and perception of work ability. Zecena Morales et al.^11^ reported that the average time until RTW for HNC patients ranged 3.6–11 months and that working in a nonprofessional role and an unsupportive work environment impeded RTW. Chiu et al.^6^ revealed that the barriers to RTW for HNC patients were poor self-perception of health, greater psychological distress, and age ≥ 50 years.

Previous studies showed that rehabilitation programs enhance physical fitness and alleviate both physical symptoms and emotional distress. Chen et al.^12^ found that a 6-month early intervention program improved muscle strength, shoulder range of motion, health-related quality of life, and the RTW rate in skilled or semi-skilled postoperative oral cancer patients. The intervention was based on phases, to include (1) early phase (8.3–30 days postsurgery): transcutaneous electrical stimulation, soft tissue mobilization and scar massage, temporomandibular joint range of motion training, active-assistive exercises for the neck and shoulder, progressive shoulder resistance exercise, functional training; (2) middle phase (1–3 months postsurgery): scar massage, soft tissue massage, joint range of motion exercise, progressive resistance exercise training, tongue and lip mobility exercise, and tongue and lip coordination training; and (3) late phase (> 3 months postsurgery): advance performance of oral, shoulder joint, and overall physical functioning according to individual performance.^12^ A study by Lo et al.^13^ of cancer survivors with job loss showed that patients who participated in a digital coaching intervention reported an increase of 12.8% in the RTW rate. The 12-week intervention, via an online eHealth app and 3 telephone health coaching sessions, involved symptom tracking, diet, exercise, peer support mindfulness, and sleep strategies.^13^ Thijs et al.^14^ found that a high-intensity physical rehabilitation program promoted a significant improvement in working hours per week and a shorter time until RTW in cancer survivors. The 18-week training program consisted of high-intensity resistance and endurance training in six parts: vertical row, leg press, bench press, pullover, abdominal crunch, and lunge.^14^

A previous study^15^ showed that RTW programs appear to minimize the barriers to RTW and strengthen psychosocial function. van Egmond et al.^15^ found that, compared to the control group, a tailored RTW program slightly improved the time until cancer survivors with job loss sustainably returned to work and resulted in a statistically nonsignificant improvement in rate of RTW, fatigue, quality of life, and participation in society for the intervention group. The program included two routes. Route 1 provided participants with three months of either job hunting for paid employment or work therapy for changing their physical conditions and ongoing benefits. Route 2 provided a coach for the same three months who helped each participant create a work profile based on an extensive inventory of the participant's wishes and needs for RTW and on the participant's work experience and capabilities.^15^

Based on these studies, we assumed that an RTW program would advance physical function and psychosocial function and reduce barriers to RTW in HNC patients at the early survivorship phase. Therefore, the present study aimed to examine the effects of an RTW program on perceived health status, barriers to RTW, fear of cancer progression, social support, physical function, and psychosocial function in HNC patients in the early survivorship phase.

## Methods

### Study design

The present study is an assessor-blinded, parallel, two-arm randomized controlled trial following the consolidated standards of reporting trials (CONSORT) statement design. It was registered in the Clinical Trial Registry under the number NCT04322695.

### Participants

A convenience sampling of HNC patients was recruited from the radiation outpatient departments of a 3700-bed medical center in northern Taiwan between August 2020 and June 2023. Patients eligible for the study met the following criteria: (1) 20–64.5 years old (retirement age in Taiwan is 65 years); (2) new diagnosis of HNC; (3) employed part-time or full-time at the time of cancer diagnosis; and (4) having completed treatment within the past 6 months and returned to work for a part-time or full-time job. The exclusion criteria were: (1) a diagnosis of mental disorder; (2) unstable systemic disease (active infection, heart disease, diabetes, or other underlying disease); and (3) physical performance < 60 on the Karnofsky Performance Status (KPS) score.^16^

### Ethical considerations

The trial was reviewed and approved by the Ethical Committee of Chang Gung Medical Foundation in Taiwan (IRB No. 201801245B0). This study followed the guidelines of the Declaration of Helsinki. Signed informed consent was obtained from all participants before recruitment.

### Randomization

Eligible patients were randomly assigned into the experimental group or the control group (RTW program vs. usual care) by a statistician using computer-generated randomization (even number: control group; odd number: experimental group). Randomization was based on a 1:1 experimental-to-control arm ratio. The outcome measurements were recorded by a research assistant.

### Intervention

The RTW program was developed based on previous studies^12^, ^13^, ^14^, ^15^^,^^17^ and a literature review.^18^ The program included two aspects: rehabilitation and the RTW program. The rehabilitation consisted of: (1) assessment and measurement of disability: identify physical ability problems and specific functional disabilities to be addressed, with the goal of providing appropriate palliative and other care for the patient's specific problems; (2) patient education: provide information related to physical disability risk factors, the side effects of surgical resection and adjuvant treatment, disability-related symptoms, and possible contraindications to treatment (eg, mobility restrictions, psychological distress, and functional disability), maintaining balance, activities to improve mobility, dietary management, and proactive care before the onset of disability; and (3) home exercise: develop a home exercise program with a focus on stretching, range of motion, and slow progression of exercise exertion over time.

The RTW program consisted of: (1) vocational counseling: evaluate patient feedback regarding the effectiveness of the program, then modify the support to better manage each patient's specific problems; (2) social welfare: provide welfare assistance for job placement and transition to employment, plus connection to a social network and other appropriate social support; (3) patient support groups: encourage patients to attend the Sunshine Social Welfare Foundation and HNC patient association; and (4) referral to shelter workshop: refer patients to the unit, which has facilities to provide vocational training. The content validity of the RTW program was evaluated by five HNC experts and preliminary tested in two HNC patients. A handbook was provided to HNC patients for guidance.

The experimental group took part in the program monthly for a period of 6 months, guided by a trained research nurse. Participants received a 60 minutes education face-to-face at the clinic, and the trained research nurse discussed concerns, addressed the problems of the patients, and tailored the intervention based on their needs. Participants received the RTW program during the trimonthly clinic follow-up visit for 15 minutes. The trained research nurse provided 10 minutes of telephone counseling biweekly as a follow-up, including a reminder to engage in home exercise and rehabilitation. The total duration was 90 minutes for the intervention and 120 minutes for the telephone sessions.

Usual care was provided to patients in the control group by the nurses of the outpatient departments during their regular clinic follow-up visits. It included patient education related to physical activity, symptom management, and monitoring for adverse reactions to treatment. Patients in the experimental group did not receive the usual care.

### Data collection

Baseline data for all measures were obtained before random assignment. Patients were measured at four time points: the baseline assessment (T0) was conducted before the RTW program (the visit to the outpatient clinic at 6 months after completing treatment); the second assessment was carried out 3 months after participating in the RTW program (T1); and subsequent assessments were performed at 6 months (T2) and 9 months (T3) after participating in the RTW program (or 9, 12, and 15 months after completing treatment, respectively).

### Measures

#### Numeric rating scale

Perceived health status was measured using the numeric rating scale (NRS), developed by McCaffery and Beebe.^19^ This scale ranges from 0 (worst health status) to 10 (best health status imaginable) points. A higher score indicates a more positive health status.^19^ Using the mean item score, ≤ 3 points is classified as mildly poor, 4–6 points is classified as moderate, and ≥ 7 points is classified as high.^20^^,^^21^ A previous study showed acceptable psychometric properties of the NRS.^22^ In this study, the Cronbach's α value for the NRS was 0.90.

#### Return to work barrier scale

Barriers to returning to work were measured using the Chinese-language version of the return to work barrier scale (RTWBS),^6^ developed by Grunfeld et al.^23^ This 17-item scale assesses two aspects of barriers to RTW: the impact of cancer and its treatment (9 items) and cognitive and emotional representations of illness (8 items). Each item is scored from 0 (strongly disagree) to 7 (strongly agree). The total possible score ranges from 0 to 119, with higher scores indicating greater barriers to RTW. A previous study has shown acceptable psychometric properties of the Chinese RTWBS.^6^ In the present study, the Cronbach α was 0.94.

#### Fear of Progression Questionnaire

The fear of cancer progression was assessed using the Chinese-language version of the Fear of Progression Questionnaire (FoP-Q-SF),^24^ developed by Mehnert et al.^25^ The FoP-Q-SF has 9 items, each scored from 1 (never) to 5 (very often), with a higher score indicating greater fear of cancer progression.^26^ The Chinese FoP-Q-SF has shown satisfactory psychometric characteristics in a HNC cancer study.^24^ In our study, the Cronbach's α value for this instrument was 0.92.

#### Medical outcomes study social support survey

Social support was assessed using the medical outcomes study social support survey (MOS SS), developed by Moser et al.^27^ The MOS SS consists of 8 items measuring two domains: emotional support (4 items) and instrumental support (4 items). Each item is scored on a scale of 0–5, and the summed score is converted into standardized scores ranging from 0 to 100, with a higher score indicating a greater level of support.^28^ A previous study has shown acceptable psychometric properties of the Chinese MOS SS.^29^ In this study, the Cronbach's α value was 0.92.

#### University of Washington Quality of Life Scale

The University of Washington Quality of Life Scale (UW-QOL) was used to assess patients' physical and psychosocial function. The UW-QOL consists of 12 items measuring two domains: physical function (6 items) and psychosocial function (6 items). Summed scores are transformed into a 0–100 scale for each domain and for total scores. Higher scores indicate greater physical or psychosocial function.^30^ The UW-QOL was translated into Chinese, and previous studies reported satisfactory psychometric properties.^31^^,^^32^ In this study, the Cronbach's α value was 0.96.

#### KPS

We used the KPS score to assess functional status. The scale ranges from 0% (death) to 100% (normal functioning).^16^

#### Sociodemographic and disease information

Sociodemographic information included age, gender, marital status, educational level, and religion. Disease information included cancer stage, tumor location, types of treatment, and radiation dosage.

### Data analysis

Analysis of the data was conducted using SPSS for Windows, version 27.0 (IBM Corp., Armonk, NY, USA). The distributions of scores were normal, which met the parametric assumption. The independent *t*-test and Chi-square test were used to examine the differences in sociodemographic, disease information, and outcome variables between the two groups at baseline. A repeated measures ANOVA was used to examine the changes in outcome variables between each time point within groups. The effects of the RTW program on outcome variables between the two groups were examined using the mixed-model repeated measure ANOVA.

The statistical power of this study was calculated using the G∗Power 3.1.9 program^33^^,^^34^ for repeated measures ANOVA. Based on a similar study,^5^ the required sample of 35 subjects in each group had an effect size of 0.08, a power of 0.08, and an α of 0.05.

## Results

### Sociodemographic and disease information by group

**Figure 1:**
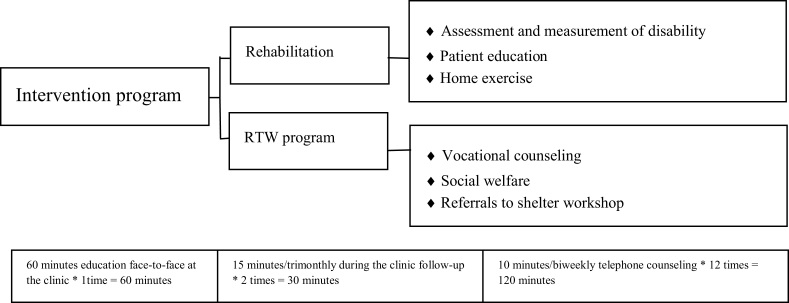
Intervention components. RTW, return to work.

**Figure 2:**
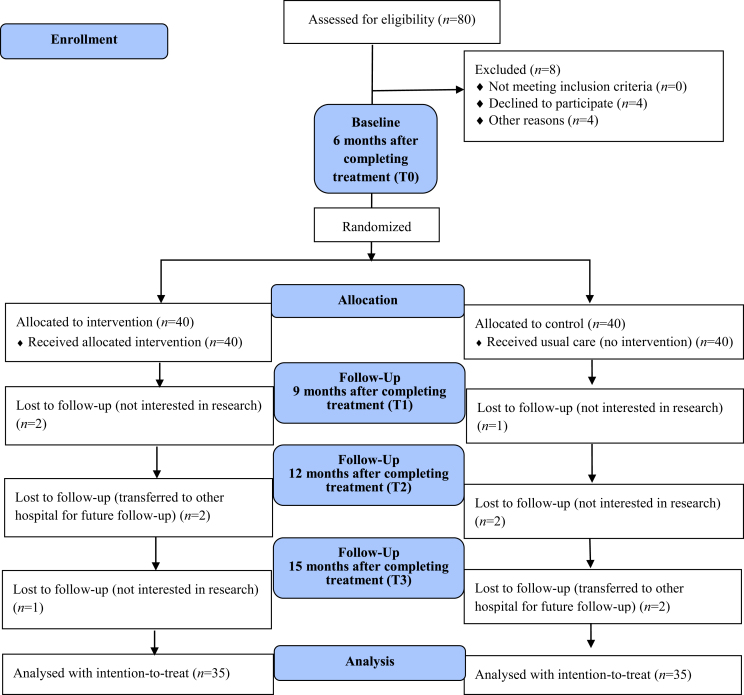
Consolidated standards of reporting trials (CONSORT) flow chart for trial recruitment.

A total of 80 eligible subjects were included. Of these, 70 subjects participated in the study, for an attrition rate of 12.5% (Fig. 1). The average age was 51.46 (standard deviation [SD] = 8.32) years for the experimental group and 48.37 (SD = 12.14) years for the control group. In both groups, most patients were male, lived with a partner, had a junior or senior high school educational level, and held Buddhism/Taoism religious beliefs. The oral cavity was the most common location of the cancer; surgery with concurrent chemotherapy and radiation therapy was the most common treatment; and most had good physical performance on the KPS. The mean radiotherapy dose was 6547.54 (SD = 366.02) cGy in the experimental group and 6434.06 (SD = 312.01) cGy in the control group (Table 1) (Fig. 2).

**Table 1:** Sociodemographic and disease characteristics by group (*N* = 70).

Variables	Experimental group (*n* = 35)	Control group (*n* = 35)	χ^*2*^*/t*	Significant
	*n* (%) / Mean (SD)	*n* (%) / Mean (SD)		
Age (years)	51.46 (8.32)	48.37 (12.14)	−1.240	0.220
Gender			2.258	0.133^a^
Male	33 (94.3)	29 (82.9)		
Female	2 (5.7)	6 (17.1)		
Marital status			0.612	0.434
Living alone	9 (25.7)	12 (34.3)		
Living with a partner	26 (74.3)	23 (65.7)		
Educational level			5.022	0.170^a^
Elementary	1 (2.9)	2 (5.7)		
Junior high	21 (60.0)	13 (37.1)		
Senior high	13 (37.1)	18 (51.4)		
College and above	0 (0)	2 (5.7)		
Religion			0.661	0.719^a^
None	3 (8.6)	5 (14.3)		
Buddhism/Taoism	28 (80.0)	27 (77.1)		
Christianity	4 (11.4)	3 (8.6)		
TNM classification			3.310	0.191^a^
II	2 (5.7)	4 (11.4)		
III	2 (5.7)	6 (17.1)		
IV	31 (88.6)	25 (71.4)		
Tumor site			3.410	0.182
Oral cavity	28 (80.0)	33 (94.3)		
Hypopharynx	6 (17.1)	2 (5.7)		
Larynx	1 (2.9)	0 (0)		
Medical treatment			1.940	0.379
Surgery + RT	13 (37.1)	18 (51.4)		
Surgery + CCRT	16 (45.7)	14 (40.0)		
CCRT	6 (17.1)	3 (8.6)		
Radiotherapy, total dose, cGy	6547.54 (366.02)	6434.06 (312.01)	−1396	0.167
Performance status	99.71 (1.69)	100.00 (0.00)	1.000	0.324

SD, standard deviation; TNM, tumor-node-metastasis; RT, radiotherapy; CCRT, concurrent chemoradiothrapy.

a Fisher's exact test.

### Comparison of baseline outcome variables

Independent *t*-tests of the differences in outcome variables between the two groups at baseline found no statistically significant differences (Table 2).

**Table 2:** Comparison of group outcomes at baseline (*N* = 70).

Variables	Experimental group (*n* = 35)		Control group (*n* = 35)		*t*	Significant
	Mean	SD	Mean	SD		
Perceived health status (NRS)	47.86	29.93	56.43	36.05	−1.082	0.283
Barriers to returning to work (RTWBS)	66.00	8.82	69.63	11.36	1.492	0.140
Fear of cancer progression (Fop-Q-SF)	24.09	5.63	23.46	6.87	−0.419	0.677
Social support (MOS SS)	68.31	16.39	63.76	19.19	−1.066	0.290
Physical function (UW-QOL)	63.95	8.71	64.69	15.27	0.248	0.805
Psychosocial function (UW-QOL)	76.24	9.21	69.74	16.91	−1.997	0.051

SD, standard deviation; NRS, numeric rating scale; RTWBS, return to work barrier scale; Fop-Q-SF, Fear of Progression Questionnaire; MOS SS, medical outcomes study social support survey; UW-QOL, University of Washington Quality of Life Scale.

### Within-group changes in outcome variables

For the two groups, perceived health status and psychosocial function increased from T0 to T3, peaking at T3, with statistically significant differences between time points. In the experimental group, barriers to RTW and fear of cancer progression slightly improved from T0 (before the RTW program) to T3 (15 months after the RTW program began), with statistical significance. In the experimental group, social support and physical function slightly increased from T0 to T2, then slightly decreased at T3, with statistical significance. In the control group, fear of cancer progression, social support, and physical function slightly increased from T0 to T1, then slightly decreased at T2 and T3. In the control group, psychosocial function slightly increased from T0 to peak at T3, with statistical significance (Table 3).

**Table 3:** Change of perceived health status, barriers to returning to work, fear of cancer recurrence, social support, physical function, and psychosocial function within group over 4 times (*N* = 70).

Variables	Group	T0^a^ (6M)	T1 (9M)	T2 (12M)	T3 (15M)	*F*	Significant	LSD^b^
		Mean (SD)	Mean (SD)	Mean (SD)	Mean (SD)			
Perceived health status (NRS)	EG	56.43 (36.05)	69.57 (36.49)	66.43 (32.05)	75.00 (30.92)	3.174	< 0.05∗	T1, T2, T3 > T0
	CG	47.86 (29.93)	50.00 (21.86)	62.14 (29.93)	63.57 (22.96)	4.996	< 0.05∗	T1, T2, T3 > T0
Barriers to returning to work (RTWBS)	EG	66.00 (8.82)	64.14 (10.02)	62.14 (9.89)	60.26 (10.80)	5.707	< 0.05∗	T0 > T1, T2, T3
	CG	69.63 (11.36)	62.37 (11.26)	60.71 (11.17)	59.42 (10.95)	28.096	< 0.001∗∗	T0 > T1, T2, T3
Fear of cancer progression (Fop-Q-SF)	EG	24.09 (5.63)	20.00 (6.17)	18.74 (6.96)	16.91 (7.20)	24.586	< 0.001∗∗	T3 > T2, T1, T0
	CG	23.46 (6.87)	25.29 (12.39)	22.74 (11.18)	22.60 (11.96)	2.085	0.122	
Social support (MOS SS)	EG	68.31 (16.39)	74.66 (13.61)	76.02 (16.18)	71.69 (15.10)	4.261	< 0.05∗	T1, T2 > T0, T3
	CG	63.76 (19.19)	74.55 (20.31)	68.16 (17.93)	65.94 (19.71)	10.075	< 0.001∗∗	T1, T2 > T0, T3
Physical function (UW-QOL)	EG	63.95 (8.71)	69.32 (11.19)	69.06 (12.44)	72.43 (15.05)	12.712	< 0.001∗∗	T3 > T0, T1, T2
	CG	64.69 (15.27)	68.38 (12.29)	71.34 (12.20)	70.01 (12.90)	5.252	< 0.05∗	T1, T2, T3 > T0
Psychosocial function (UW-QOL)	EG	76.24 (9.21)	83.04 (13.11)	86.01 (11.36)	86.69 (10.88)	17.977	< 0.001∗∗	T1, T2, T3 > T0
	CG	69.74 (16.91)	75.95 (18.14)	79.34 (15.50)	80.91 (16.57)	9.894	< 0.001∗∗	T1, T2, T3 > T0

∗*P* < 0.05.

∗∗*P* < 0.01.

SD, standard deviation; EG, experimental group; CG, control group; NRS, numeric rating scale; RTWBS, return to work barrier scale; Fop-Q-SF, Fear of Progression Questionnaire; MOS SS, medical outcomes study social support survey; UW-QOL, University of Washington Quality of Life Scale.

a Baseline: before the return to work program.

b LSD: least significance difference; comparison based on Bonferonni adjustment.

### Effects of the return to work program on the outcome variables

In the experimental group, health status, barriers to RTW, fear of cancer recurrence, social support, physical function, and psychosocial function improved significantly from pre-test to posttest (ie, T0 to T3). For both groups, health status and psychosocial function improved significantly from T0 to T3. After 9 months, the experimental group had a significantly larger group-by-time interaction effect than the control group for barriers to RTW and fear of cancer recurrence (Table 4).

**Table 4:** Mixed model: repeated measures of health status, barriers to returning to work, fear of cancer recurrence, social support, physical function, and psychosocial function by group (*N* = 70).

Variables	Pretest^a^ Mean (SD)	Posttest^b^ Mean (SD)	Between-groups, *F*_b_ (*P*)^c^	Within-times, *F*_w_ (*P*)^d^	Interaction, *F*_in_ (*P*)^e^
Perceived health status (NRS)			4.191 (< 0.05∗)	6.547 (< 0.05∗)	1.285 (0.281)
EG	56.43 (36.05)	75.00 (30.92)			
CG	47.86 (29.93)	63.57 (22.96)			
Barriers to returning to work (RTWBS)			0.002 (0.965)	26.129 (< 0.001∗∗∗)	3.480 (< 0.05∗)
EG	66.00 (8.82)	60.26 (10.80)			
CG	69.63 (11.36)	59.42 (10.95)			
Fear of cancer progression (Fop-Q-SF)			3.445 (0.068)	11.880 (< 0.001∗∗∗)	7.594 (< 0.05∗)
EG	24.09 (5.63)	16.91 (7.20)			
CG	23.46 (6.87)	22.60 (11.96)			
Social support (MOS SS)			1.522 (0.222)	11.450 (< 0.001∗∗∗)	2.180 (0.100)
EG	68.31 (16.39)	71.69 (15.10)			
CG	63.76 (19.19)	65.94 (19.71)			
Physical function (UW-QOL)			0.001 (0.974)	14.552 (< 0.001∗∗∗)	1.635 (0.192)
EG	63.95 (8.71)	72.43 (15.05)			
CG	64.69 (15.27)	70.01 (12.90)			
Psychosocial function (UW-QOL)			4.783 (< 0.05∗)	25.180 (< 0.001∗∗∗)	0.080 (0.948)
EG	76.24 (9.21)	86.69 (10.88)			
CG	69.74 (16.91)	80.91 (16.57)			

EG, experimental group; CG, control group; NRS, Numeric Rating Scale; RTWBS, Return to Work Barrier Scale; Fop-Q-SF, Fear of Progression Questionnaire; MOS SS, Medical Outcomes Study Social Support Survey; UW-QOL, University of Washington Quality of Life Scale.

∗*P* < 0.05.

∗∗*P* < 0.01.

∗∗∗*P* < 0.01.

a Measured before the return to work program.

b Measured at 9-months after receiving the tailored transitional return to work program (15 months after completing treatment).

c *F*_b:_ the *F* value between groups comparison.

d *F*_w:_ the *F* value within pre- and posttest.

e *F*_in:_ the *F* value of the interaction of between groups and within pretest and posttest.

## Discussion

### Implications of study results

After participating in this study, the experimental group had a higher level of perceived health status than the control group at 9 months. This finding supports the results of Chen et al.^12^ who revealed that an early intervention program improved muscle strength, shoulder range of motion, and health-related quality of life in oral cancer survivors. These findings suggest that a tailored home-based exercise program contributes to physical activity and movement and improves overall health and fitness to facilitate an increase in the patient's feeling of well-being and lessen the barriers to RTW.

The experimental group showed a greater increase in psychosocial function than the control group, as well as a higher level of social support, although the difference between groups was not statistically significant. These findings are dissimilar to those of a previous report, which found that a tailored RTW program did not improve quality of life or participation in society.^15^ However, the studies differed in the working status of the participants. Our subjects completed treatment within the past 6 months and returned to work, while the cancer survivors in the study by van Egmond et al.^13^ had experienced job loss. To strengthen the effect on social participation, the RTW program can include social activities such as participation in support groups (eg, Sunshine Social Welfare Foundation and the Chinese Association for Cancer Care).

The RTW program did not significantly reduce barriers to RTW for patients in the experimental group compared to those in the control group. In both groups, barriers to RTW decreased from T0 (before the RTW program) to T3 (9 months after participating in the RTW program) and were lowest at T3, with the difference being statistically significant. These findings differed from those of a prior study.^14^ However, the studies differed in the time since completing treatment. Our participants had completed treatment within 6 months and were in the early survival phase, while participants in the study by Thijs et al.^14^ had completed treatment more than 6 months previously. Thijs et al.^14^ also found that cancer survivors who received a high-intensity physical rehabilitation program had improvements in the number of hours worked per week and in the time until RTW. Findings from this current study also reflect the fact that most Taiwanese HNC patients are labor workers and may experience long-term side effects of treatment. These findings suggest that healthcare professionals need to assess and record the barriers to RTW and educate patients to help them find relevant resources.

At 9 months, barriers to RTW and fear of cancer recurrence were lower in participants in the RTW program than in those in the control group. Patients who participated in the RTW program were those whose fear of cancer progression demonstrating a shift from T0 to T3, a peak at T0 to a low at T3 (9 months after participating in the RTW program), with statistically significant differences. This finding supports the results of a prior study.^35^ Deuning-Smit et al.^35^ identified a decline in fear of cancer from diagnosis to 6 months posttreatment in newly diagnosed HNC patients. Because the differences were significant between groups at 9 months, it appears the RTW program helped these patients, who acquired more support from employers and friendly a work environment as they transitioned from the early survival and maintenance phases. Thus, it is important to assess inner thoughts and feelings regarding the cancer progression and educate patients by sharing their reflections on the fear of cancer recurrence.

### Clinical implications

In clinical care, healthcare providers can provide patients with an RTW program guide to help them manage the adverse effects of treatment, promote physical and psychosocial function, and reduce the barriers to RTW for HNC patients during the early survival phase.

### Limitations

There are several limitations to this study. First, in this study, we recruited participants who were returning to work and did not include patients with job losses. Future studies are recommended to expand to populations with different work statuses and compare the effects of an RTW program on these outcome variables. Second, this study did not assess the working environment and working conditions, nor the effect that these variables may have on barriers to RTW. Studies are needed to involve workplace factors and identify the effects of different workplace characteristics on barriers to RTW. Finally, among the types of work in this study, we did not consider barriers to RTW. Thus, future studies should recruit subjects engaged in a variety of job types to examine the correlation between physical conditions and barriers to RTW by job type.

## Conclusions

The results of this study indicate HNC patients who received an RTW program had significantly greater perceived health status and better psychosocial function than those who received usual care. Moreover, the experimental group had significantly improved fear of cancer progression, social support, and physical function from T0 to T3, compared to the control group. However, the intervention did not significantly reduce the barriers to RTW or the fear of cancer progression, nor did it significantly improve social support or physical function.

## Acknowledgments

The authors gratefully acknowledge the contribution of the patients who participated in the study. The authors would like to thank Convergence CT for assistance with English editing during development of the manuscript.

## CRediT author statement

YLC, BSH and SCC designed the study. BSH, CYL, CFC and YLC involved in data collection and analysis. BSH and SCC prepared the manuscript. All authors were granted complete access to all the data in the study, with the corresponding author bearing the final responsibility for the decision to submit for publication. The corresponding author affirms that all listed authors fulfill the authorship criteria and that no others meeting the criteria have been omitted.

## Declaration of competing interest

The authors declare no conflict of interest.

## Funding

10.13039/100012553This research was supported by fundings received from Chang Gung Memorial Hospital Research Program (CMRP, Grant Nos. CMRPF1K0021, CMRPF1K0022, and NMRPF3M0071). The funders had no role in considering the study design or in the collection, analysis, interpretation of data, writing of the report, or decision to submit the article for publication.

## Ethics statement

All procedures performed in studies involving human participants were in accordance with the ethical standards of the University of Wisconsin Health Sciences IRB (IRB No. 201801245B0) and with the 1964 Helsinki Declaration and its later amendments or comparable ethical standards. All participants provided written informed consent and voluntarily participated in the study.

## Data availability statement

The data that support the findings of this study are available from the corresponding author. Restrictions apply to the availability of these data, which were used under license for this study. Data are available from the authors with the permission of Chang Gung Memorial Hospital Research Program (CMRP).

## Declaration of Generative AI and AI-assisted technologies in the writing process

No AI tools/services were used during the preparation of this work.
